# Disordering of Starch Films as a Factor Influencing the Release Rate of Biologically Active Substances

**DOI:** 10.3390/polym15102303

**Published:** 2023-05-13

**Authors:** Ekaterina Podgorbunskikh, Timofei Kuskov, Anna Matveeva, Artem Ulihin, Aleksey Bychkov, Igor Lomovskiy, Yuliya Polienko

**Affiliations:** 1 Institute of Solid State Chemistry and Mechanochemistry SB RAS, 18 Kutateladze Str., 630090 Novosibirsk, Russia; t.kuskov@g.nsu.ru (T.K.); matveeva@solid.nsc.ru (A.M.); ulikhin@solid.nsc.ru (A.U.); bychkov.a.l@gmail.com (A.B.); lomovsky@solid.nsc.ru (I.L.); 2 Department of Natural Sciences, Novosibirsk State University, 2 Pirogova Str., 630090 Novosibirsk, Russia; 3 Department of Business, Novosibirsk State Technical University, 20 Prospekt K. Marksa, 630073 Novosibirsk, Russia; 4 Laboratory of Nitrogen Compounds, N. N. Vorozhtsov Novosibirsk Institute of Organic Chemistry SB RAS, 9 Lavrentiev Ave., 630090 Novosibirsk, Russia; polienko@nioch.nsc.ru

**Keywords:** crystal structure, mechanochemistry, disordering, starch films, biodegradable packaging material, drug delivery, nitroxide radical, EPR

## Abstract

The release of a spin probe (nitroxide radical) from polymer films was studied by electron paramagnetic resonance (EPR). The films were fabricated from starch having different crystal structures (A-, B-, and C-types) and disordering degrees. Film morphology (analysis of the scanning electron microscopy (SEM)) depended on the presence of dopant (nitroxide radical) to a larger extent rather than on crystal structure ordering or polymorphic modification. The presence of nitroxide radical led to additional crystal structure disordering and reduced the crystallinity index from the X-ray diffraction (XRD) data. Polymeric films made of amorphized starch powder were able to undergo recrystallization (crystal structure rearrangement), which manifested itself as an increase in crystallinity index and phase transition of the A- and C-type crystal structures to the B-type one. It was demonstrated that nitroxide radical does not form an individual phase during film preparation. According to the EPR data, local permittivity of starch-based films varied from 52.5 to 60.1 F/m, while bulk permittivity did not exceed 17 F/m, which demonstrates that local concentration of water is increased in the regions near the nitroxide radical. The mobility of the spin probe corresponds to small stochastic librations and is indicative of the strongly a mobilized state. The application of kinetic models made it possible to find out that substance release from biodegradable films consists of two stages: matrix swelling and spin probe diffusion through the matrix. Investigation of the release kinetics for nitroxide radical demonstrated that the course of this process depends on the type of crystal structure of native starch.

## 1. Introduction

Despite all the advances in implementing biodegradable materials, synthetic polymers are currently used most frequently for food packaging; the demand for packaging increases in direct proportion to the rise in the world’s population [1,2]. The use of composites consisting of biodegradable and synthetic polymers is not an optimal trend in the development of packaging materials, since it results in formation of microplastics [3,4]. Only by implementing fully biodegradable polymers that can decompose in the environment (including animal and human bodies) can the stress imposed on the environment be reduced and, ultimately, also make it safer for humans.

Natural polysaccharides such as cellulose, starch, and chitosan are the most promising feedstock for producing composite materials [5,6,7]. To be efficiently used as food packaging, polymers need to have tailored barrier properties, mechanical characteristics, water and gas permeability, as well as intensity of interaction with the packaged object (e.g., during the release of active substances such as antioxidants, preservatives, pigments, and flavoring agents encapsulated in the film) that would be stable over time and under different environmental conditions [8,9,10,11,12].

Furthermore, biodegradable polymeric carrier materials are becoming increasingly common in pharmaceutics for designing capsules, films, aerogels, and emulsions to be administered through the oral, buccal, sublingual, ocular, and transdermal routes. The applicability of polymers for biomedical purposes is determined by the drug release kinetics [13,14], which largely depends on the physical properties of the polymeric matrix (particle shape and size, porosity, and swelling capacity) [15]. In terms of chemical properties, the drug release rate is expected to be related to the polymer structure; thus, linear and amorphous polymers are supposed to be dissolved and hydrolyzed faster than branched and crystalline ones [16]. Therefore, obtaining information about variations in the structure and properties of the polymer matrix at the macro- and micro-levels still remains relevant.

Electron paramagnetic resonance (EPR) is a promising method for studying the kinetics and the mechanisms of release of encapsulated substances. EPR offers remarkable opportunities for focusing on the internal properties of materials (free volume, local concentration of substances, polymer chain mobility, polarity of the local environment, etc.), as well as spatial distribution of paramagnetic molecules (spin probes) in the matrix [17,18,19]. Thus, the use of nitroxide radicals as a spin probe allows one to observe the mobility of dopant molecules at different stages of their release from the polymer matrix [15,20].

Starch is fully biodegradable; its degradation in the body gives rise to non-toxic monomers. Starch-based materials are widely used for food packaging and as biodegradable medical products [21]. These materials exhibit excellent oxygen barrier properties, but insufficient moisture resistance and poor mechanical properties, so their use is limited. In order to eliminate these drawbacks, starch can be subjected to physicochemical modification or mixed with other natural biopolymers to produce composite materials [22].

The properties of starch-based materials largely depend on the structure and the ratio of amylose and amylopectin, which form the amorphous-crystalline structure of the starch granule. Starches from different sources depending on the packing of double helices are subdivided into three polymorphic modifications of crystal structure: the A-, B-, and C-type having different patterns of X-ray diffraction [23]. One of the ways to influence the crystalline structure of natural polymers is mechanical treatment, which provides changes in properties of the materials [24]. X-ray diffraction analysis is widely used to determine the crystal structure and its changes due to modification [25,26,27].

This study aimed to produce biodegradable polysaccharide-based materials with different types and degrees of crystal structure ordering suitable for manufacturing packaging and drug release materials with tailored properties. The main hypothesis tested in the presented work was that the crystal structure is responsible both for the nature of the amorphization/recrystallization processes and for the release of encapsulated substance from the polymer matrix.

## 2. Materials and Methods

### 2.1. Materials

**Figure 1:**
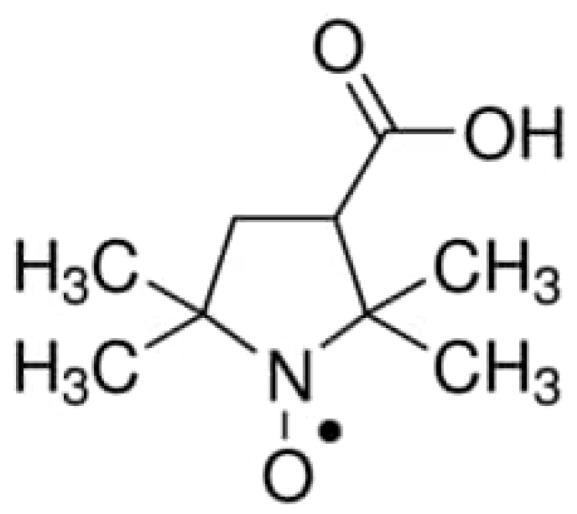
A spin probe; nitroxide radical 3-carboxy-PROXYL.

Corn starch (State Standard GOST 32159-2013, top grade, Garnec OJSC, Vladimir, Russia), potato starch (State Standard GOST R 53876-2010, top grade, Garnec OJSC, Vladimir, Russia), and tapioca starch (State Standard GOST 32159-2013, top grade, Garnec OJSC, Vladimir, Russia), ethanol (95.0%, Konstanta Farm M LLC, Moscow, Russia) and glycerol (≥99.0%, SIGMA-Aldrich, Moscow, Russia) were used in this study. Nitroxide radical 3-carboxy-2,2,5,5-tetramethyl-1-pyrrolidine-1-oxyl (3-carboxy-PROXYL, Novosibirsk Institute of Organic Chemistry, SB RAS) is shown in Figure 1.

The probe was chosen due to its hydrophilic nature. According to the standard approach, hydrophilicity is defined as the octanol/water partition coefficient. It has been shown in Refs. [28,29] that the octanol/water partition coefficient for carboxy-PROXYL is several orders of magnitude lower than that of the widely used Tempol spin probe.

### 2.2. Mechanical Treatment (MT)

Mechanical treatment (MT) of starch was carried out in a water-cooled AGO-2 (ISSCM SB RAS, Novosibirsk, Russia) laboratory planetary ball mill (grinding media: steel balls 5 mm in diameter, weighing 200 g; grinding body acceleration, 200 m/s^2^; nominal motor power 1.1 kW) using a procedure similar to that described in Ref. [24]. The weight of starch was 10 g and the treatment duration were 30 and 600 s.

### 2.3. Film Preparation

Suspensions (3 wt.%) of native starches were subjected to gelatinization at 80 °C for 30 min. Nitroxide radical at a concentration of 0.04 mg/mL (2 × 10^−5^ mol/100 mL) was then added to the gellified starch suspension. The resulting solution was kept at room temperature under stirring at 300 rpm for 30 min, and vacuum degassing was performed. The degassed solutions were poured into round silicone molds 35 mm in diameter (0.36 g/cm^2^) and dried in a drying oven at 35 °C for 24 h to obtain films.

### 2.4. X-ray Diffraction Analysis (XRD)

The structural properties of native starches, mechanically treated starches, native starch films, and starch films with 3-carboxy-PROXYL were characterized by X-ray diffraction (XRD) analysis on a D8 Advance diffractometer (Bruker, Karlsruhe, Germany) with monochromatic CuKα radiation in the Bragg–Brentano reflection geometry. The step size was 0.0195°. The analysis was performed in a broad range of 2θ angles (3 ÷ 70°) at a voltage of 40 kV and current of 40 mA. The X-ray wavelength was 1.5406 Å.

The crystallinity index of the study objects was calculated using the previously proposed modification of the Nara and Komiya method [25,30]. A smoothened curve connecting the peak baselines was superimposed onto the recorded XRD pattern. The area above the smoothed curve corresponded to the crystalline portion of starch, while the area under the curve corresponded to the amorphous one.

The crystallinity index (CrI) was calculated as the ratio between the area of the crystalline phase and the total area under the XRD curve using the formula:

CrI = S(cr.phase)/S(total) × 100%,(1)

where S(cr.phase) is the area of the crystalline phase and S(total) is the total area under the XRD curve.

### 2.5. The Specific Surface Area (SSA)

The specific surface area (SSA) of the samples was determined according to thermal desorption of nitrogen on a Sorptometer M instrument (Catakon, Novosibirsk, Russia) using the Brunauer–Emmett–Teller equation (the BET method) [31].

### 2.6. Particle Size

The particle size of native and mechanically treated starches was measured on a CAMSIZER X2 optical analyzer (Retsch GmbH, Haan, Germany) equipped with a compressed air dispersion module (pressure, 50 kPa); the detection threshold was 0.8–8000 µm. The average particle size was determined by image (1000 images) analysis in compliance with ISO 13322-2:2006. Furthermore, we determined a sphericity parameter b/l, which is the ratio between the minimal and the maximal inscribed chords [32].

### 2.7. Scanning Electron Microscopy (SEM)

The particle morphologies of native starches, mechanically treated starches, native starch films, and starch films with 3-carboxy-PROXYL were characterized by scanning electron microscopy (SEM) on a TM-1000 microscope (Hitachi, Tokyo, Japan) at an accelerating voltage of 15 kV; the sample surface was preliminarily coated with gold on a JFC-1600 magnetron sputter coater (Jeol, Tokyo, Japan) (sputtering duration, 40 s; ion current, 30 mA; sputtered layer thickness, 10 nm).

### 2.8. Moisture Content

Moisture content in native and mechanically treated starches and starch-based films was determined on a WPS 50 SX moisture analyzer (Radwag, Radom, Poland); the samples were heated at 130 °C using a halogen lamp as a heating element until the sample weight became constant.

### 2.9. Film Thickness

Film thickness was measured using an MK 0–25 mm micrometer (Calibre, Moscow, Russia) (State Standard GOST 6507-90). The film thickness at 10 different spots was measured for each sample.

### 2.10. Dielectric Spectroscopy

Permittivity of the films prepared from native and mechanically treated starches with 3-carboxy-PROXYL added was measured using a Precision LCR Meter HP4284A analyzer of electrophysical properties operating at frequencies of 1 MHz and 100 Hz. The permittivity was calculated according to the measured data with allowance for the geometric parameters of the samples.

### 2.11. EPR Spectroscopy

**Figure 2:**
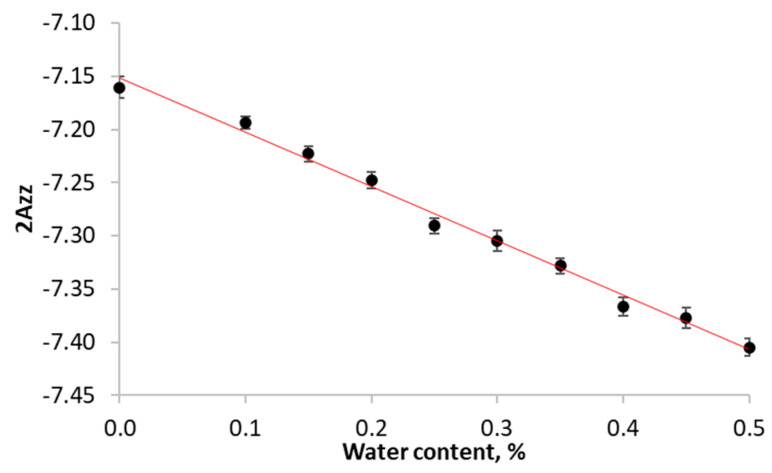
Calibration curve 2Azz of solvent polarity of frozen 0.1 μM aqueous-glycerol solutions of 3-carboxy-PROXYL. Equation y = −7.1476 − 0.5221 × x with R^2^ coefficient of 0.9915.

A Spinscan X stationary EPR spectrometer (Adani, Minsk, Belarus) was used for EPR measurements. Room-temperature experiments with fluid solutions were conducted in 50 μL capillary tubes (BlauBrand IntraMark, Cluj-Napoca, Romania). All other experiments were carried out in NMR tubes (OD, 5 mm) (Wilmad Glass, Vineland, NJ, USA). For convenience, starch-based films were cut into fragments sized 3 × 3 mm^2^. Low-temperature measurements were conducted at −196 °C in a quartz Dewar. The 2Azz value was obtained as the distance between the maxima of low-field and high-field components. In order to improve the accuracy, the low-field and high-field components were fitted by a Gaussian function. The calibration of 2Azz on solvent polarity was carried out at series of frozen 0.1 μM water–glycerol solutions of 3-carboxy-PROXYL (Figure 2). EPR acquisition parameters for each case were chosen in accordance with the standard recommendations [33].

Parameters of hyperfine structure for 3-carboxy-PROXYL in starch films were obtained by fitting the −196 °C EPR spectra. We used EasySpin 5.2.35 Software (https://easyspin.org/, accessed on 24 March 2023) [34], namely the rigid limit approximation.

The 2Azz value in the starch films was then recalculated to the local permittivity using the obtained empirical equation:

*ε* = 2*Azz* × 73.1 − 480.47, (2)

where *ε* is the permittivity, F/m; 2*Azz* is the distance between the maxima of low-field and high-field components.

### 2.12. Release Experiments

Starch-based films fragments sized 3 × 3 mm^2^ were placed into 5 mL tubes containing water-ethanol solution (77% ethanol). It turned out that in water, 3-carboxy-PROXYL was released from the film too quickly, making it difficult to properly monitor the process. On the contrary, in a 95% ethanol solution, the radical was almost never released from the matrix, so it was also impossible to adequately monitor the process. Similar observations of the release behavior of encapsulated red cabbage anthocyanin extract from polymer films of modified starch were shown in ref. [35]. Therefore, a solution with an ethanol concentration of 77% was selected, which provides the optimal rate of the process. During the experiment, the sample was thermostabilized and shaken on a TS-100 thermo-shaker (BIOSAN, Riga, Latvia). The conditions were maintained constant (stirring at 1010 rpm at 30 °C) for 7 h. The shacking rate was chosen to prevent any concentration gradient in the solution. A temperature of 30 °C, which is close to room temperature, was chosen to exclude the methodological errors caused by temperature instability. Sampling was carried out after 0, 3, 5, 10 15, 20, 25, and 30 min; then after 30 min, every 10 min; after 2 h, every 20 min; and after 6 h, every 30 min. The concentration of 3-carboxy-PROXYL in the solution in each probe was measured by EPR.

### 2.13. Release Kinetics Models

Some of the kinetic models previously reported in Refs. [36,37,38] were used for kinetics description.

The zero-order kinetic equation:

C = k_0_ × t, (3)

where C is the concentration of drug in the drug molecule; k_0_ is the constant of apparent dissolution rate; and t is time.

The first-order kinetic model:

(4) $$C\left(t\right)=C_{\infty }\left(1-e^{-\mathrm{kt}}\right),$$

where C is the concentration of drug in the drug molecule; k is the first-order release constant; and t is time.

The Higuchi kinetics model:

(5) $$C=K_{H}\times \sqrt{t},$$

where C is the concentration of drug in the drug molecule; K_H_ is the release constant of Higuchi; and t is time.

The Hixson–Crowell kinetic model:

(6) $$\sqrt[{3}]{1-\frac{C_{i}}{C_{0}}}=1-K_{\beta }\times t,$$

where C_i_/C_0_ represents the fraction of drug dissolved on time t; K_β_ is the release constant; and t is time.

The Power-law kinetic model:

(7) $$\frac{C_{i}}{C_{0}}=k\times t^{n},$$

where C_0_ is the amount of drug at the equilibrium state; C_i_ is the amount of drug released over time t; k is the constant of incorporation of structural modifications and geometric characteristics of the system (also considered the release rate constant); n is the exponent of release (related to the drug release mechanism) in function of time t; and t is time.

The Peppas–Sahlin kinetic model:

(8) $$\frac{C_{i}}{C_{\infty }}=K_{\mathrm{Fick}}\times t^{m}+K_{\mathrm{degr}}\times t^{2m},$$

where C_i_ is the amount of the drug released at time instant t; C_∞_ is the amount released at the infinite t; m is constant (m = 0.45); K_Fick_ is the Fick’s constant; K_degr_ is the degradation constant; and t is time.

### 2.14. Statistical Analysis

The results are presented as the mean ± standard deviation (SD). The intergroup differences in the groups of starch sources were tested for statistical significance using an unpaired Student’s *t*-test. The intergroup differences between starch sources were tested for statistical significance using the one-way analysis of variance (ANOVA). The *p*-value was considered non statistically significant at *p* ≥ 0.05; and significant, at *p* < 0.05.

## 3. Results and Discussion

### 3.1. Native and Mechanically Treated Starch

According to IUPAC definition [39], crystal structure disordering is any deviation from the ideal three-dimensional regularity of the crystal structure. Here, we use the complex approach for the description of crystal structure disordering. X-ray powder diffraction is most widely employed as a clear, easy to interpret, and routine method to establish the crystal structure and observe the processes of amorphization of substances, including native polymers [25,40,41,42]. The changes in the surface properties of powders can be assessed using the thermal desorption of gases (determination of the specific surface area). The morphology of materials can be directly captured by scanning electron microscopy (SEM), but it is here also supplemented with the CAMSIZER X2 optical analyzer data.

First, the polymorphic modifications of each starch were identified by X-ray diffraction [25]. The XRD patterns of corn starch contain diffraction peaks at 2θ = 15.0°, 17.0°, 17.9°, and 22.9°, which are characteristic of A-type starch. The XRD patterns of potato starch contain diffraction peaks at 2θ = 5.6°, 15.1°, 17.2°, 19.7°, 22.2°, 24.0°, and 26.4°, which are characteristic of B-type starch. The polymorphic modification of C-type tapioca starch has a mixed crystal structure consisting of the B-type (the granule core) and A-type (the peripheral regions of the granule) starches. Diffraction peaks at 2θ = 5.6°, 15.2°, 17.1°, 18.2°, and 22.8° are characteristic of tapioca starch.

**Figure 3:**
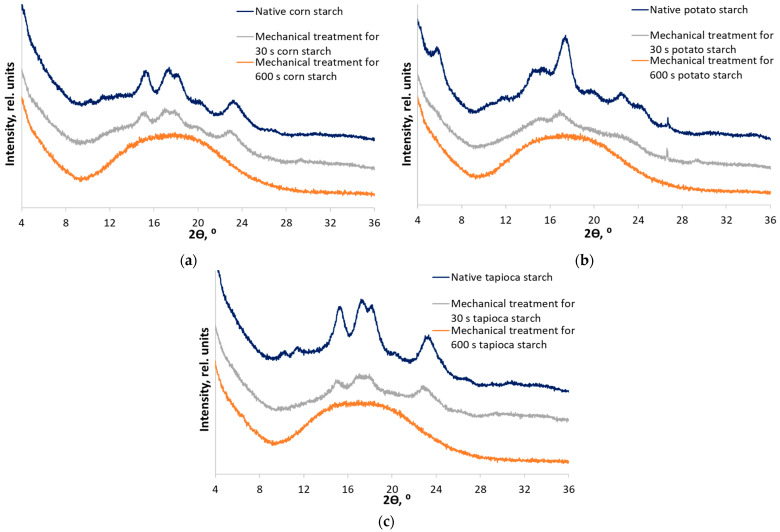
X-ray diffraction patterns of native samples and samples subjected to mechanical treatment for 30 and 600 s: (**a**) corn starch (A-type); (**b**) potato starch (B-type); and (**c**) tapioca starch (C-type).

Native starch (NS) samples were subjected to mechanical treatment for 30 and 600 s. The choice of treatment duration was based on our previous works [24,43]. Thus, starches treated for 30 s (MT30) differed noticeably from native starches, while not being completely amorphous. Mechanical treatment for 600 s (MT600) results in known amorphous starch samples. Indeed, the corresponding XRD patterns (Figure 3) indicate stages of disordering of the crystal structure. Table 1 shows alterations in all the measured physicochemical characteristics of mechanically treated starches. The crystallinity indices of native starches before mechanical treatment have significant differences (ANOVA, *F_stat_* = 42.3, *p*-value = 2.89 × 10^−4^). There are no significant differences in the values of the crystallinity index of partially disordered starches (MT30) (ANOVA, *F_stat_* = 2.3, *p*-value = 0.178). It is notable that disordering is strongly non-linear in time: the intermediate stage occurs already after processing for 30 s, but much more time is required for complete amorphization.

**Figure 4:**
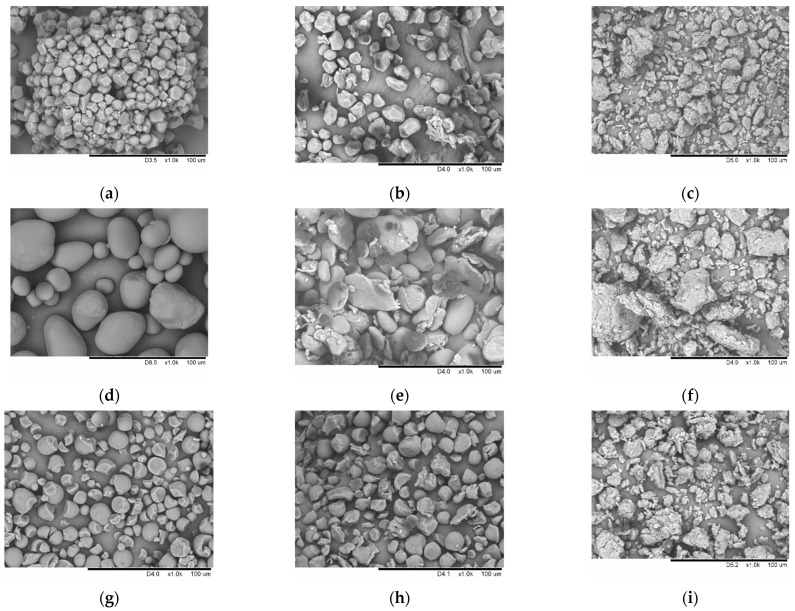
The micro images of native samples and samples subjected to mechanical treatment for 30 and 600 s: corn starch (**a**–**c**), potato starch (**d**–**f**), and tapioca starch (**g**–**i**).

Changes occurring at the macroscopic level were monitored by SEM. Corn starch granules (A-type) are irregular polyhedra with concave faces (Figure 4a). Potato starch granules (B-type) have a spherical, elliptical, or irregular shape (Figure 4d). Tapioca starch granules are predominantly spherical and have a smooth surface (Figure 4g). After mechanical treatment for 30 s, the granule shape of all starch types changes due to the impact shearing of the grinding bodies (Figure 4b,e,h). The long-term mechanical treatment for 600 s results in the complete destruction of all the types of starch granules and the formation of secondary aggregates from smaller particles (Figure 4c,f,i). Therefore, comparison of the XRD and SEM data demonstrates that the mechanical treatment of starch is accompanied by the destruction of the macrostructure of starch grains and the amorphization of its microstructure.

Table 1 summarizes the values characterizing the size, shape, and surface of starch samples after mechanical treatment. Native potato starch has the largest particle size (ANOVA, *F_stat_* = 11,668.0, *p*-value = 1.69 × 10^−11^). It is worth mentioning that mechanical treatment results in crushing as a result of high mechanical loads; it is accompanied by a decline in sphericity and stable particle aggregation (Figure 4c,f,i; Table 1). All particle size distributions were unimodal. Starch becomes amorphized and the specific surface area increases with the rising time of mechanical treatment.

In terms of its specific surface area, native potato starch (B-type) differs significantly from native tapioca starch (C-type), while native corn starch (A-type) does not differ significantly from B- and C-type (ANOVA, *F_stat_* = 7.0, *p*-value = 0.027). It can be observed that partial disordering of B-type (MT30 potato starch) provided the lowest SSA compared to A- and C-type starches (MT30 corn and tapioca starch) (ANOVA, *F_stat_* = 43.0, *p*-value = 2.77 × 10^−4^). The specific surface area after mechanical treatment for 600 s does not differ for all starch types (ANOVA, *F_stat_* = 1.0, *p*-value = 0.422).

Thus, a set of nine samples with different degrees of crystal structure disordering was obtained: three stages of disordering for three types of starch.

### 3.2. Film Preparation

**Figure 5:**
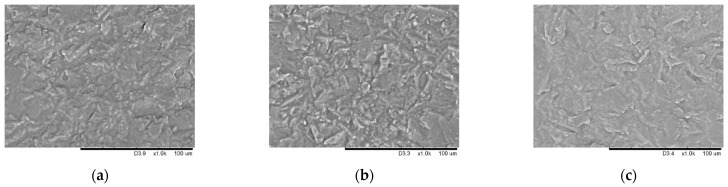

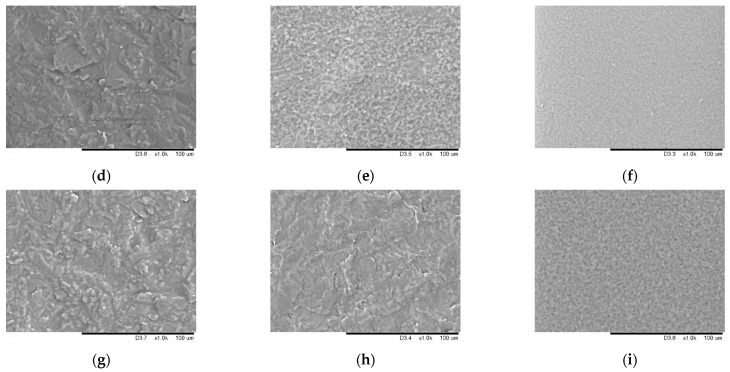
Micro images of native starch films and films prepared from mechanically treated starch for 30 and 600 s, with 3-carboxy-PROXYL nitroxide radical added: corn (**a**–**c**), potato (**d**–**f**), and tapioca starch (**g**–**i**).

Films were prepared from starch of different ordering degrees using the casting method. The films based on native starch were semi-transparent and textured, the ones made of starch subjected to mechanical treatment for 30 s were less transparent and had a smoother surface. Films prepared from completely amorphous starch (after mechanical treatment for 600 s) were characterized by the most smooth and homogeneous surface and transparency (data not shown). Figure 4 shows the micro images of the films with 0.04 mg/mL nitroxide radical added. SEM micro images of films based on native starch have a textured surface (Figure 5a,d,g). The ones prepared from mechanically treated starch have a homogeneous regular microstructure without any inclusions (Figure 5c,f,h). Micro images of the films without the spin probe added are shown in Supplementary Materials (Figure S1).

**Figure 6:**
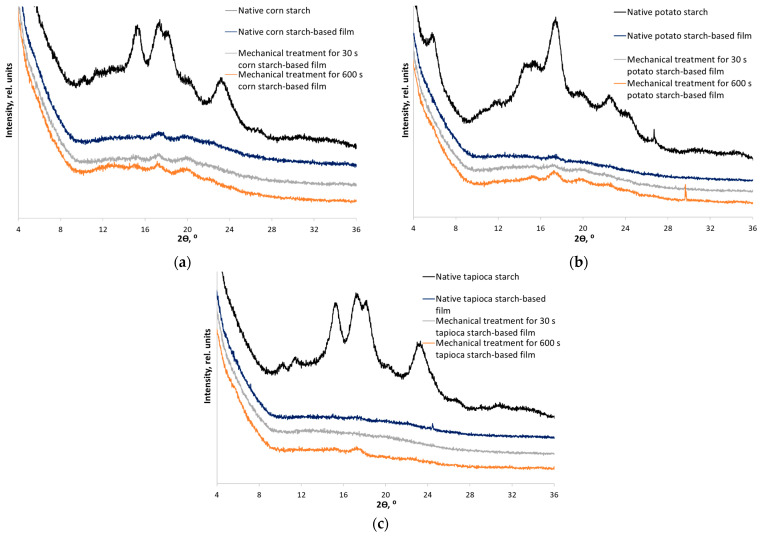
X-ray diffraction patterns of the films based on native starch and starch mechanically treated for 30 and 600 s with 3-carboxy-PROXYL nitroxide radical added: corn (**a**), potato (**b**), and tapioca starch (**c**).

X-ray diffraction analysis of films based on starches with different ordering degrees indicates that the crystallinity index of the resulting materials after gelatinization was significantly reduced (Figure 6 and Table 2). However, the films with 0.04 mg/mL nitroxide radical added are characterized by lower crystallinity compared to those without the spin probe (Supplementary Materials Table S1).

It means that the spin probe (nitroxide radical) acts as a plasticizer preventing the formation of perfect crystals. The interaction of the carboxyl group of 3-carboxy-PROXYL with OH-groups of starch stabilizes the amorphous phase. Thus, the formation of a large number of hydrogen bonds during film preparation predominantly prevents crystallization [44,45] The XRD patterns of the films with and without the spin probe are compared in Supplementary Materials (Figure S2). The structure is least ordered for all the films prepared from partially disordered starch within type (subjected to mechanical treatment for 30 s). When films are formed from completely amorphized starch (subjected to mechanical treatment for 600 s), recrystallization and transition of the A- and C-type crystal structure to the B-type were observed (Figure 6). The observed phenomenon is also known as recrystallization and is well-studied for other amorphous-crystalline polymers such as cellulose [46,47]. The crystal structure and crystallinity index of the films after recrystallization do not differ (ANOVA, *F_stat_* = 4.5, *p*-value = 0.064).

Thickness of the films (Table 2) based on partially disordered starch (subjected to mechanical treatment for 30 s) is noticeably higher compared to that of the films prepared from mechanically amorphized starches. Thickness of the films based on native starch and completely amorphized starch is not significantly different. In the case of corn starch, mechanical treatment for 30 s did not significantly altered thickness—only an increasing trend (*p*-value < 0.2) was observed. The thickness of the films without the spin probe added are shown in Supplementary Materials (Table S1). It is assumed that the 3D polymer chain of native starch disrupted during mechanical treatment is capable of denser packing. When considering the effect of starch type on the thickness of films prepared from completely amorphous starch, there are no differences between corn starch (A-type) and potato starch (B-type) (ANOVA, *F_stat_* = 13.06, *p*-value > 0.002).

One can see that crystal structure disordering in a polymer has a significant effect on the characteristics (polymorphic modification and crystallinity, homogeneity, thickness, and moisture content) of the end products for edible packaging. Recrystallization (crystal structure rearrangement) becomes available for the fully disordered samples subjected to mechanical treatment for 600 s, which manifests itself as an increase in the degree of crystallinity and transition of polymorphic modification to the B-type starch.

### 3.3. EPR Spectroscopy of Starch Films

#### 3.3.1. Spin Probe Behavior in Films

In order to monitor the processes related to the encapsulation of substances, we prepared films with the 3-carboxy-PROXYL probe added. This spin probe was selected because it is hydrophilic, non-toxic, and stable both in the solid phase and in solutions.

**Figure 7:**
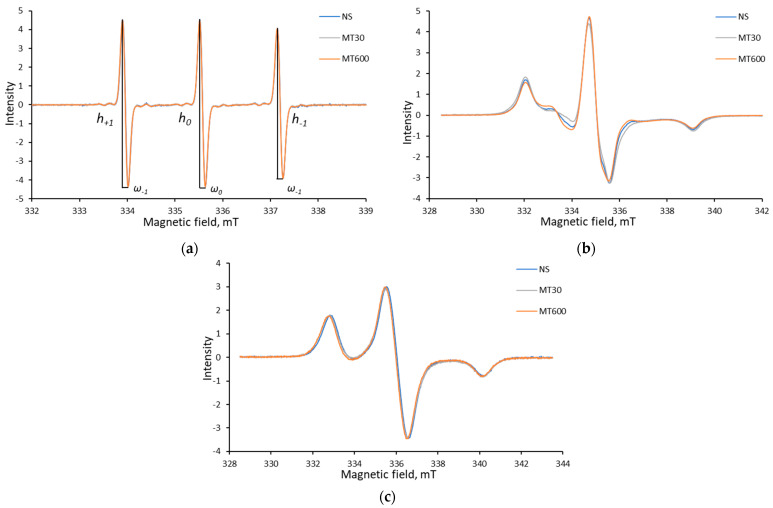
The characteristic EPR spectra of 3-carboxy-PROXYL nitroxide radical for potato starch as an example: in the liquid state (**a**); in the film at room temperature (**b**); and the stabilized spectrum at −196 °C (**c**).

Figure 7 shows the EPR spectra of the spin probe at different stages of its incorporation into the films. All film spectra are compared in Supplementary Materials (Figures S3 and S4). Probe incorporation into the film changes mobility and, therefore, alters the spectral shape. One can see that at room temperature, the probe exists in the film in an intermediate state, which is more similar to solid phases. Meanwhile, the integral intensity of the spectra remains constant, thus indicating that 3-carboxy-PROXYL is stable during film preparation.

At the gelatinization stage of all starch types, the spectra of the probe correspond to free rotation. According to the known approach in Ref. [48] we can calculate the rotational correlation times τ_c_:

(9) $$\tau_{c}=6.5\times 10^{-10}\times \omega_{0}\left(\sqrt{\frac{h_{0}}{h_{-1}}}-1\right),$$

where *w*_0_ is the linewidth of the central EPR line expressed in Gauss; *h*_0_ and *h*_−1_ are heights of the central and high-field lines, respectively. The obtained τ_c_ values are listed in Table 3. We can further recalculate the rotation correlation times to local viscosity η using the Stokes–Einstein equation:

(10) $$D=\frac{\mathrm{kT}}{6\pi \eta R},$$

where the diffusion coefficient D is defined from τ_c_ as, $R^{2}={D\tau }_{c}$; and R is the effective radius of the spin probe. One can estimate the rotation radius as being half of the maximal distance between the atoms. In our case, the R of the spin probe is near 3.2 Å, therefore, at room temperature T = 298 K (25 °C) for typical value τ_c_ = 40 ps local viscosity η is equal to 4.2 × 10^−6^ m^2^/s. The value exceeds the water viscosity at room temperature 4 times, 1.0 × 10^−6^ m^2^/s, but is significantly lower than the macroviscosity of the starch samples during the gelatinization stage. Disagreement between macro- and microviscosities is a typical feature of polymer solutions [49], but here it also indicates that there are no stable interactions between a starch macromolecule and spin probe.

Next, no exchange-narrowed EPR line belonging to crystalline 3-carboxy-PROXYL is observed in the spectra of the films; i.e., the spin probe is distributed in the films without forming a separate phase during film preparation.

In order to quantify nitroxide mobility in starch films, we used the model of small stochastic librations (small-angle motion without full rotation) [50]. Indeed, the value ΔA_zz_ (the difference between the Azz value in liquid nitrogen and at current temperature) is a criterion of transition from libration to the rotation model. It is considered that before ΔA_zz_ ≈ 0.5 mT is reached, rotations are forbidden, and it is fully implemented for our systems because in our case ΔA_zz_ ≈ 0.2 mT. In accordance with Ref. [50], we can calculate the amplitude of libration angle α:

(11) $$\Delta A_{\mathrm{zz}}=\left(A_{\mathrm{zz}}-A_{\mathrm{yy}}\right)\langle \alpha^{2}\rangle .$$

Here, A_zz_ and A_yy_ are two of the three principal components of hyperfine tensor, defined from frozen spectra by simulation using the widely used EasySpin software [34]. For all the films, the obtained angle α value is ~15°. Similar behavior was observed earlier, for example, in polystyrene films near −133–(−113) °C [51] and in deep eutectic solvent choline chloride–thiourea at −33–(−23) °C [52]. Therefore, the motion of 3-carboxy-proxyl is strongly restricted in starch film. The obtained values of angle α for each film are listed in Table 3. One can see that the films obtained from completely amorphous starch are characterized by the lowest rigidity of the starch matrix.

Moreover, EPR allows one to measure local dielectric permittivity (polarity of the medium near spin probe) in order to compare it with the macroscopic one. This was done by comparing the EPR spectra for films and water–glycerol solutions (50–100 wt.% of glycerol) at −196 °C. The permittivity of the solutions with different glycerol–water ratios over a broad range is well known [53]; therefore, we could use these solutions as a calibration scale. Using the 2Azz value (the distance between the high-field and low-field peaks in frozen samples) of nitroxides as a descriptor of polarity of the medium has been previously demonstrated [54,55]. Here, we plot the known *ε* of water–glycerol solutions onto the measured 2Azz value according to Equation (2).

The 2Azz value in starch films was then recalculated to the local permittivity. Obtained ε values lay in the range of 52.5–60.1 F/m (Table 3).

The comparison of permittivity determined from the EPR and dielectric spectroscopy data showed that polarity of the local environment of the nitroxide radical is higher than that for the polymer material as a whole.

The permittivity ε of the films measured by dielectric spectroscopy is strongly non-linear with respect to moisture content, being indicative of the interplay between the bound and the free state of water [56]. In our measurements, the dielectric constants did not exceed 17 F/m at the maximum film moisture content of 12%, which is consistent with the findings obtained earlier [56]. The capacity of the condenser and polymer films was investigated in two frequency ranges determining the effect of polarization and sample geometry.

This means that nitroxide radical is preferentially distributed over the polymer regions with an increased water content (20–40%). This is important because the content and state of water (free or bound) plays a crucial role in the exchange of encapsulated substances from the carrier matrix. Here, for the films based on fully amorphized potato and tapioca starch, the local water concentration is noticeably higher than that for films from native starches.

Films based on starch subjected to mechanical treatment for 30 s have similar *ε* for all the types of polymorphic modifications (ANOVA, *F_stat_* = 2.86, *p*-value > 0.134). Mechanical treatment for 600 s leads to local polarity, which is noticeably higher than that for films made from native starches, but row *ε* (potato starch) > *ε* (tapioca starch) > *ε* (corn starch) initially measured for films from native starches (NS), being reliably reproduced for the MT600 samples.

#### 3.3.2. Release of Spin Probes from Films

The kinetics of spin probe transfer from the carrier matrix into the solution was studied by measuring its concentration in the solution. In order to minimize starch swelling in aqueous solutions and control the time of radical transfer from the film, the optimal composition of the water–ethanol mixture simulating the conditions of carrier matrix degradation was determined (data not shown) and a further experiment was carried out at the optimal concentration (77% ethanol in water).

The time dependence between the total intensity of EPR peaks was plotted (Figure 7).

**Figure 8:**
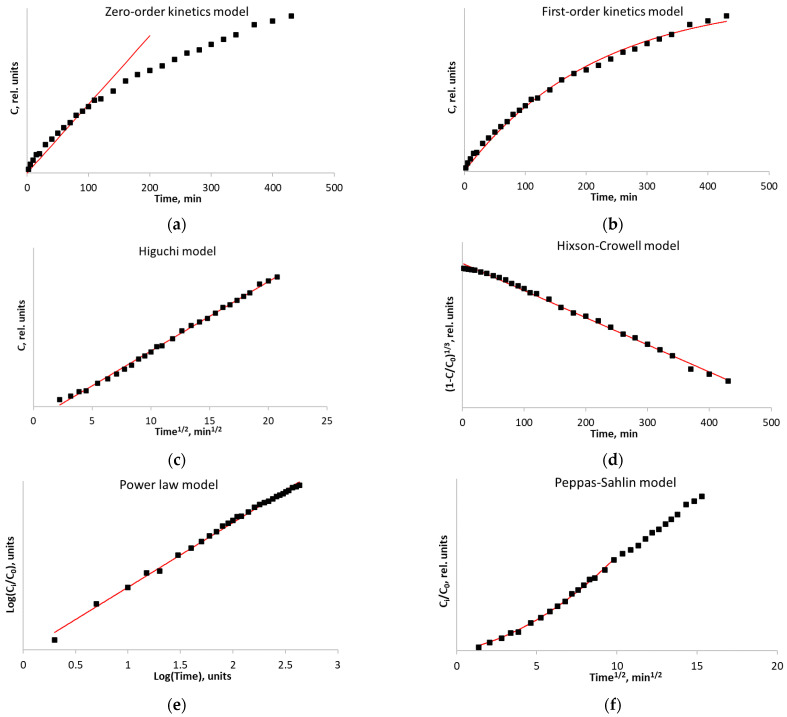
The kinetics of spin probe transfer from the film (for native corn starch) with nitroxide radical added: the zero-order kinetic model (**a**); the first-order kinetic model (**b**); the Higuchi kinetic model (**c**); the Hixson–Crowell kinetic model (**d**); the Power law kinetic model (**e**); and the Peppas–Sahlin kinetic model (**f**). ▪—Experimental data.

Figure 8a shows that the radical transfer kinetics are not fitted by the zero-order kinetic equation (Equation (3)).

The first-order kinetic model (Equation (4)) with an R^2^ coefficient of 0.9942 (Figure 7b) fits the release kinetics with an appreciably high estimation accuracy, thus indicating that the experimental conditions have been properly selected. Such a behavior of the kinetic curve indicates that the release is directly proportional to the radical concentration gradient; the process runs in the kinetic mode.

The Higuchi kinetics model (Equation (5)) describes the kinetics based on radical diffusion from the matrix, and the particle size remains unchanged during the diffusion. The mismatch between the kinetic data and the Higuchi model (Figure 8c) indicates that the matrix is changed during the radical release.

The Hixson–Crowell kinetic model (Equation (6)), which is based on an assumption that the release process is limited by the swelling rate, describes the kinetics (Figure 8d) with the coefficient of determination (R^2^) of 0.9963, which demonstrates that degradation, swelling, or carrier matrix relaxation make a significant contribution during probe release from the polymer film.

An analysis using the Power-law model (Equation (7)) in rectifying logarithmic coordinates shows (Figure 7e) that the initial probe release stage is fitted by a straight line. The final stage is also fitted by a straight line, but with a smaller slope angle. This behavior indicates that both processes, matrix swelling and diffusion through the matrix, contribute almost equally to the process of substance release.

Therefore, the description of the process using the kinetic model proposed by Peppas and Sahlin (Equation (8)) [37] seems most plausible (Figure 8f); this model combines two types of processes: Fick’s diffusion of the probe from the polymer matrix and the rapid probe release at the boundary of polymer matrix swelling.

Indeed, this model is most suitable for reliable fitting of the release kinetics of 3-carboxy-PROXYL radical from starch-based films with R^2^ coefficient > 0.9965. A similar analysis was performed for all the kinetic curves. Table 4 summarizes the Fick constants (K_Fick_), the degradation constants (K_degr_), and coefficients (R^2^).

The equilibrium concentration C_i_ achieved in the solution during the release of 3-carboxy-PROXYL has no regularity with the duration of mechanical treatment. The equilibrium concentration of films prepared from native corn starch (NS) differs significantly from the NS of potato and tapioca starch films (ANOVA, *F_stat_* = 303.3, *p*-value = 7.29 × 10^−7^). K_Fick_, a parameter describing the diffusion rate of the probe in the matrix, increases for native starch-based films as a series of A- < B- = C-type (ANOVA, *F_stat_* = 25.0, *p*-value = 0.0012). It can be observed that partial disordering of B-type (MT30 potato starch-based film) provided a higher diffusion rate compared to A- and C-type (MT30 corn and tapioca starch-based film) (ANOVA, *F_stat_* = 23.5, *p*-value = 0.002). Mechanical treatment for 600 s (MT600) does not lead to significant changes in K_Fick_ for all types of starches (ANOVA, *F_stat_* = 1.6, *p*-value = 0.279). Mechanical treatment (within the same type) does not lead to significant changes in K_Fick_ for all types of starches. The constant K_degr_, which describes the interaction between the matrix and the solvent, for films based on native corn starch (A-type) differs significantly from that of native tapioca starch (C-type), while films based on native potato starch (B-type) do not differ significantly from films A- and C-type (ANOVA, *F_stat_* = 9.8, *p*-value = 0.013). The films obtained from MT30 and MT600 do not have significant differences for all types of starches.

The release kinetics of encapsulated substances for a completely disordered starch structure becomes independent of the type of initial structure and physicochemical parameters of the native starches.

The crystal structure of native starch plays an enormous role in controlling the characteristics of biodegradable materials. No explicit correlations between the nitroxide radical release rate and the crystallinity index for the main types of starch powders (A-, B-, and C-types) were revealed in this study.

## 4. Conclusions

Films have been prepared from starches with a different crystal structure (the initial polymorphic modifications A-, B-, and C-types) and disordering degrees; the resulting films have been characterized. All the films prepared from partially disordered starch have the lowest crystallinity index. Recrystallization (crystal structure rearrangement) occurs when films are prepared from completely disordered starch, manifesting itself as an increase in crystallinity index and phase transition to the B-type structure.

Mechanical pretreatment of starch affects the film morphology when dopants are added. Films based on native starch are characterized by a textured surface; mechanical disordering of starch powder contributes to the formation of a homogeneous regular microstructure without any inclusions. The morphology of the films without spin probe added was independent of the disordering degree of starch.

Therefore, nitroxide radical acts as an plasticizing agent, resulting in lower crystallinity compared to the films not carrying the spin probe. Any additive will act as a plasticizer, the spin probe has no specificity in this case. Since this study focuses on drug release, special emphasis was placed on how the encapsulated substance changes the properties of the matrix. In this sense, it would be incorrect to study pure films without adding a probe and then conclude that doped films should have the same properties.

It has been demonstrated that during film preparation (the incorporation of substances), nitroxide radical is distributed over the films without forming an individual phase. Moreover, EPR measurement allows one to sense the local environment of an encapsulated substance. First, the mobility of nitroxide radical in the resulting films was determined using the model of small stochastic librations; the angle α value for all the films was ~15°, which means strongly restricted motions. Second, the local permittivity of starch-based films varies from 52.5 to 60.1 F/m, which indicates a noticeable increase in the local concentration of water near the dopant.

The use of kinetic models revealed that the release of substances from biodegradable films is described by two processes: matrix swelling and spin probe diffusion through the matrix. Investigation of the release kinetics (the nitroxide radical being used as an example) showed that the course of this process depends on the type of crystal structure of native starch. However, further research is needed to gain a deeper understanding of the processes involved in the design and control over the operation properties of the biodegradable polysaccharide materials that are suitable for developing packaging and drug release materials.

## Figures and Tables

**Table 1:** Physicochemical parameters of native and mechanically treated starches.

Sample	Average Particle Size, µm	Sphericity, b/l	Crystallinity Index, %	SSA ^1^, m^2^/g
Corn starch				
NS ^2^	19.0 ± 0.1	0.798 ± 0.001	36 ± 2	0.7 ± 0.1
MT30 ^3^	16.2 ± 0.1	0.784 ± 0.001	25 ± 2	1.3 ± 0.1
MT600 ^4^	23.8 ± 0.1	0.772 ± 0.001	AS ^5^	1.6 ± 0.1 ^b^
Potato starch				
NS	28.4 ± 0.1	0.780 ± 0.001	29 ± 1	0.5 ± 0.1
MT30	28.2 ± 0.1 ^a^	0.747 ± 0.001	22 ± 1	0.6 ± 0.1 ^a^
MT600	29.7 ± 0.1	0.753 ± 0.001	AS	1.5 ± 0.1
Tapioca starch				
NS	16.6 ± 0.1	0.839 ± 0.001	42 ± 2	0.8 ± 0.1
MT30	16.6 ± 0.1 ^a^	0.793 ± 0.001	24 ± 2	1.2 ± 0.1
MT600	27.5 ± 0.1	0.758 ± 0.001	AS	1.5 ± 0.1 ^b^

Data are presented as mean ± SD. ^1^ SSA—specific surface area; ^2^ NS—native starch; ^3^ MT30—starch after mechanical treatment for 30 s; ^4^ MT600—starch after mechanical treatment for 600 s; ^5^ AS—amorphous sample. ^a^ No significant differences compared to NS samples (*p*-value ≥ 0.05). ^b^ No significant differences compared to MT30 samples (*p*-value ≥ 0.05).

**Table 2:** The key physicochemical characteristics of films based on native starch and starch with different disordering degrees (with 3-carboxy-PROXYL added).

Starting Material	Type	Crystallinity Index, %	Film Thickness, µm	Moisture Content, %
Corn starch-based film				
NS ^1^	A-type	14 ± 1	69 ± 7	7.9 ± 0.1
MT30 ^2^	A-type	10 ± 1	85 ± 6 ^a^	9.3 ± 0.1
MT600 ^3^	B-type	15 ± 1 ^a^	63 ± 5 ^a^	10.9 ± 0.1
Potato starch-based film				
NS	B-type	19 ± 1	59 ± 3	7.5 ± 0.1
MT30	B-type	15 ± 1	84 ± 4	8.0 ± 0.1
MT600	B-type	18 ± 2 ^a,b^	64 ± 6 ^a^	9.3 ± 0.1
Tapioca starch-based film				
NS	C-type	11 ± 1	47 ± 3	12.4 ± 0.1
MT30	C-type	7 ± 1	69 ± 4	10.5 ± 0.1
MT600	B-type	15 ± 1	49 ± 2 ^a^	9.4 ± 0.1

Data are presented as mean ± SD. ^1^ NS—native starch; ^2^ MT30—starch after mechanical treatment for 30 s; ^3^ MT600—starch after mechanical treatment for 600 s. ^a^ No significant differences compared to NS samples (*p*-value ≥ 0.05). ^b^ No significant differences compared to MT30 samples (*p*-value ≥ 0.05).

**Table 3:** The correlation times of 3-carboxy-PROXYL nitroxide radical at the gelatinization stage, the mean angle of its stochastic librations in starch films, and EPR-measured local permittivity in films with different structure ordering degrees.

Starting Material	Correlation Time (τ_c_), ps	Angle of Stochastic Librations, °	Permittivity, F/m
Corn starch-based film			
NS ^1^	50 ± 2	10.88 ± 0.02	52.5 ± 0.2
MT30 ^2^	40 ± 2	11.86 ± 0.02	56.9 ± 0.2
MT600 ^3^	42 ± 2 ^b^	13.31 ± 0.02	56.2 ± 0.3 ^b^
Potato starch-based film			
NS	44 ± 2	13.15 ± 0.02	56.0 ± 0.3
MT30	32 ± 2	12.97 ± 0.02	57.3 ± 0.3
MT600	38 ± 2 ^a,b^	14.42 ± 0.02	60.1 ± 0.3
Tapioca starch-based film			
NS	52 ± 2	12.93 ± 0.02	54.5 ± 0.3
MT30	40 ± 2	13.06 ± 0.02	57.4 ± 0.3
MT600	41 ± 2 ^b^	13.91 ± 0.02	58.9 ± 0.4

Data are presented as mean ± SD. ^1^ NS—native starch; ^2^ MT30—starch after mechanical treatment for 30 s; ^3^ MT600—starch after mechanical treatment for 600 s. ^a^ No significant differences compared to NS samples (*p*-value ≥ 0.05). ^b^ No significant differences compared to MT30 samples (*p*-value ≥ 0.05).

**Table 4:** The constants of the release kinetics of 3-carboxy-PROXYL nitroxide radical from a polymeric film with different structure ordering degrees.

Starting Material	Type	C_i_, rel.Units	K_Fick_, s^−1/2^ × 100	K_degr_, s^−1^ × 1000	R^2^
Corn starch-based film					
NS ^1^	A-type	6356 ± 164	1.7 ± 0.2	2.2 ± 0.2	0.9994
MT30 ^2^	A-type	6257 ± 220 ^a^	2.4 ± 0.2 ^a^	2.0 ± 0.2 ^a^	0.9979
MT600 ^3^	B-type	7801 ± 244	2.4 ± 0.3 ^a,b^	2.2 ± 0.4 ^a,b^	0.9967
Potato starch-based film					
NS	B-type	10361 ± 278	2.7 ± 0.2	2.0 ± 0.2	0.9985
MT30	B-type	3948 ± 95	3.3 ± 0.3 ^a^	2.4 ± 0.3 ^a^	0.9974
MT600	B-type	6224 ± 148	2.1 ± 0.2 ^a,b^	2.8 ± 0.3 ^a,b^	0.9985
Tapioca starch-based film					
NS	C-type	10653 ± 230	2.7 ± 0.2	1.5 ± 0.2	0.9984
MT30	C-type	9132 ± 189	2.0 ± 0.2 ^a^	2.2 ± 0.2 ^a^	0.9965
MT600	B-type	7062 ± 210	2.1 ± 0.2 ^a,b^	2.2 ± 0.2 ^a,b^	0.9976

Data are presented as mean ± SD. ^1^ NS—native starch; ^2^ MT30—starch after mechanical treatment for 30 s; ^3^ MT600—starch after mechanical treatment for 600 s. ^a^ No significant differences compared to NS samples (*p*-value ≥ 0.05). ^b^ No significant differences compared to MT30 samples (*p*-value ≥ 0.05).

## Data Availability

Not applicable.

## Supplementary Materials

The following supporting information can be downloaded at: https://www.mdpi.com/article/10.3390/polym15102303/s1, Figure S1: Micro images of native starch films and films prepared from starch treated mechanically for 30 and 600 s: corn (a–c), potato (d–f), and tapioca starch (g–i).; Figure S2: X-ray diffraction patterns of the films based on native starch and starch mechanically treated for 30 and 600 s with and without 3-carboxy-PROXYL added: corn (a–c), potato (d–f), and tapioca starch (g–i).; Figure S3: The characteristic EPR spectra of 3-carboxy-PROXYL nitroxide radical for corn starch: in the liquid state (a); in the film at room temperature (b); and the stabilized spectrum at −196 °C (c).; Figure S4: The characteristic EPR spectra of 3-carboxy-PROXYL nitroxide radical for tapioca starch: in the liquid state (a); in the film at room temperature (b); and the stabilized spectrum at −196 °C (c).; Table S1: The key physicochemical characteristics of films based on native starch and starch with different disordering degrees (without 3-carboxy-PROXYL added).
