# Supplementary material for: Disordering of Starch Films as a Factor Influencing the Release Rate of Biologically Active Substances
Source: Polymers (Basel). 2023 May 13;15(10):2303. doi: 10.3390/polym15102303 (PMC10222158; doi:10.3390/polym15102303)
Supplement: Supplementary file 1 [file polymers-15-02303-s001.zip › polymers-2335134-supplymentary.pdf]

polymers

MDPI

Disordering of Starch Films as a Factor Influencing the Release Rate of Biologically Active Substances

Ekaterina Podgorbunskikh [1,*], Timofei Kuskov [1,2], Anna Matveeva [1], Artem Ulihin [1], Aleksey Bychkov [1,3], Igor Lomovskiy [1] and Yuliya Polienko [4]

[1] Institute of Solid State Chemistry and Mechanochemistry SB RAS, 18 Kutateladze Str., 630090 Novosibirsk, Russia; t.kuskov@g.nsu.ru (T.K.); matveeva@solid.nsc.ru (A.M.); ulikhin@solid.nsc.ru (A.U.); bychkov.a.l@gmail.com (A.B.); lomovsky@solid.nsc.ru (I.L.)
[2] Department of Natural Sciences, Novosibirsk State University, 2 Pirogova Str., 630090 Novosibirsk, Russia
[3] Department of Business, Novosibirsk State Technical University, 20 Prospekt K. Marksa, 630073 Novosibirsk, Russia
[4] Laboratory of Nitrogen Compounds, N. N. Vorozhtsov Novosibirsk Institute of Organic Chemistry SB RAS, 9 Lavrentiev Ave., 630090 Novosibirsk, Russia; polienko@nioch.nsc.ru
* Correspondence: podgorbunskikh@solid.nsc.ru

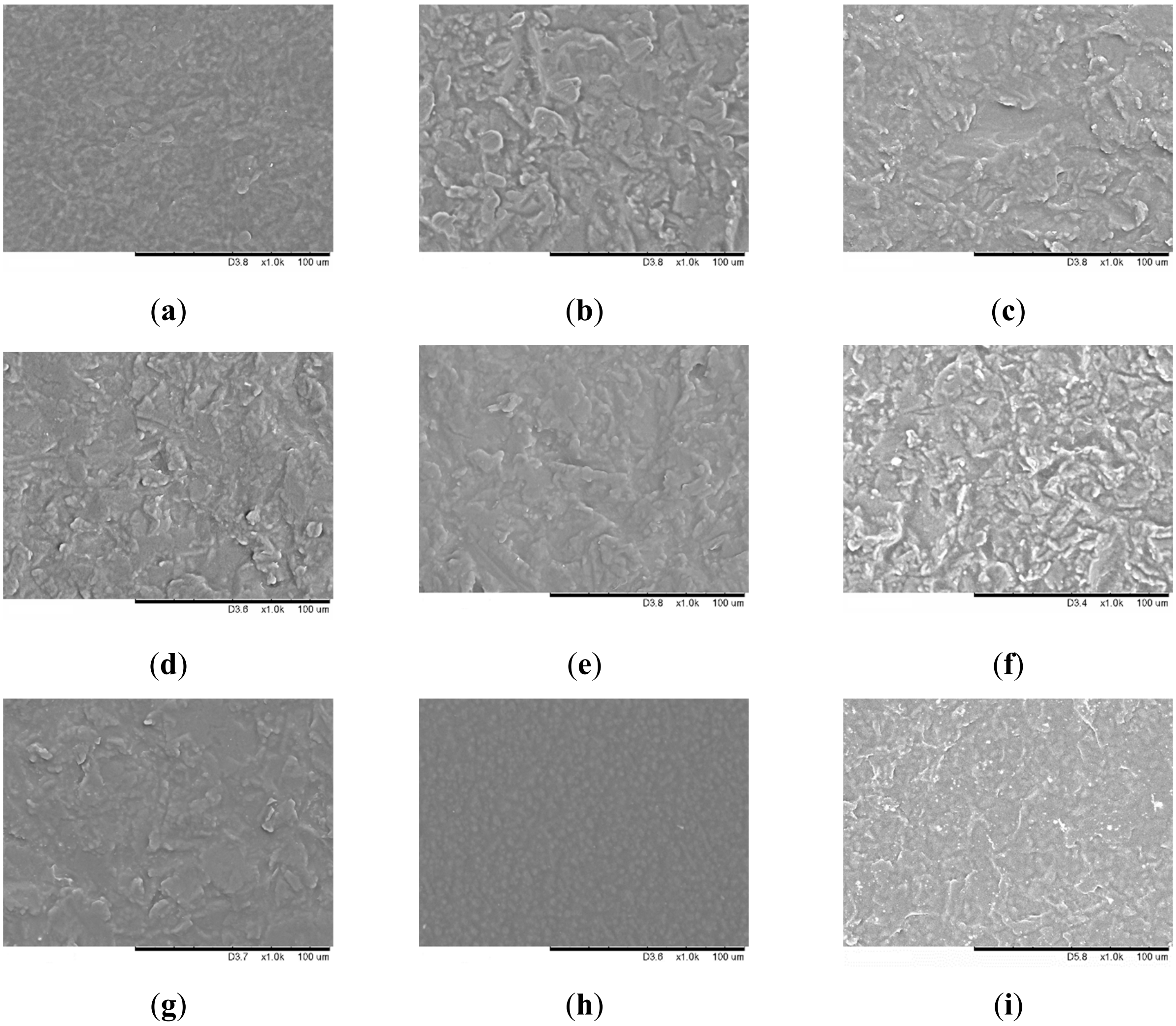

Figure S1. Micro images of native starch films and films prepared from starch treated mechanically for 30 and 600 s: corn (a–c), potato (d–f), and tapioca starch (g–i).

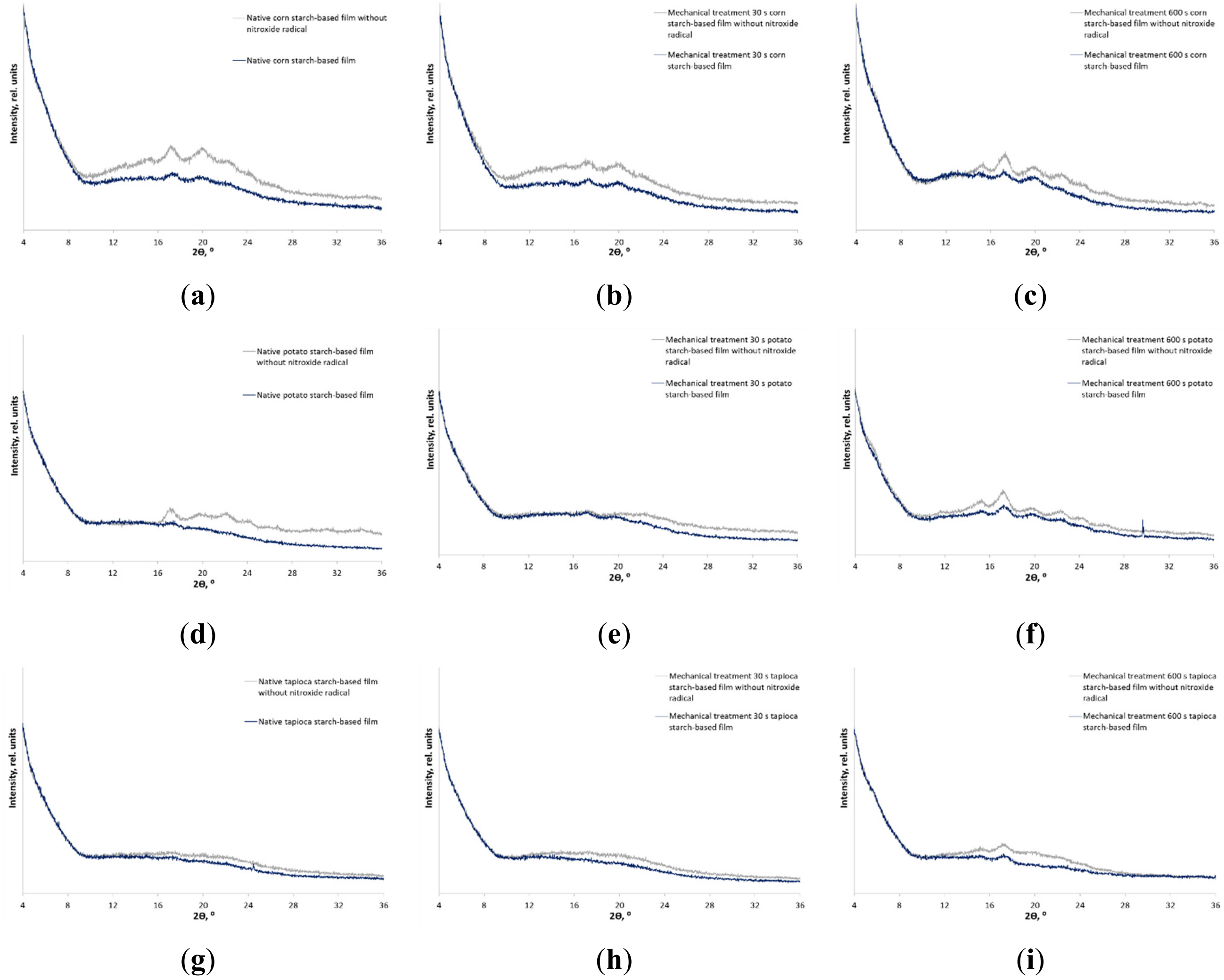

Figure S2. X-ray diffraction patterns of the films based on native starch and starch mechanically treated for 30 and 600 s with and without 3-carboxy-PROXYL added: corn (a–c), potato (d–f), and tapioca starch (g–i).

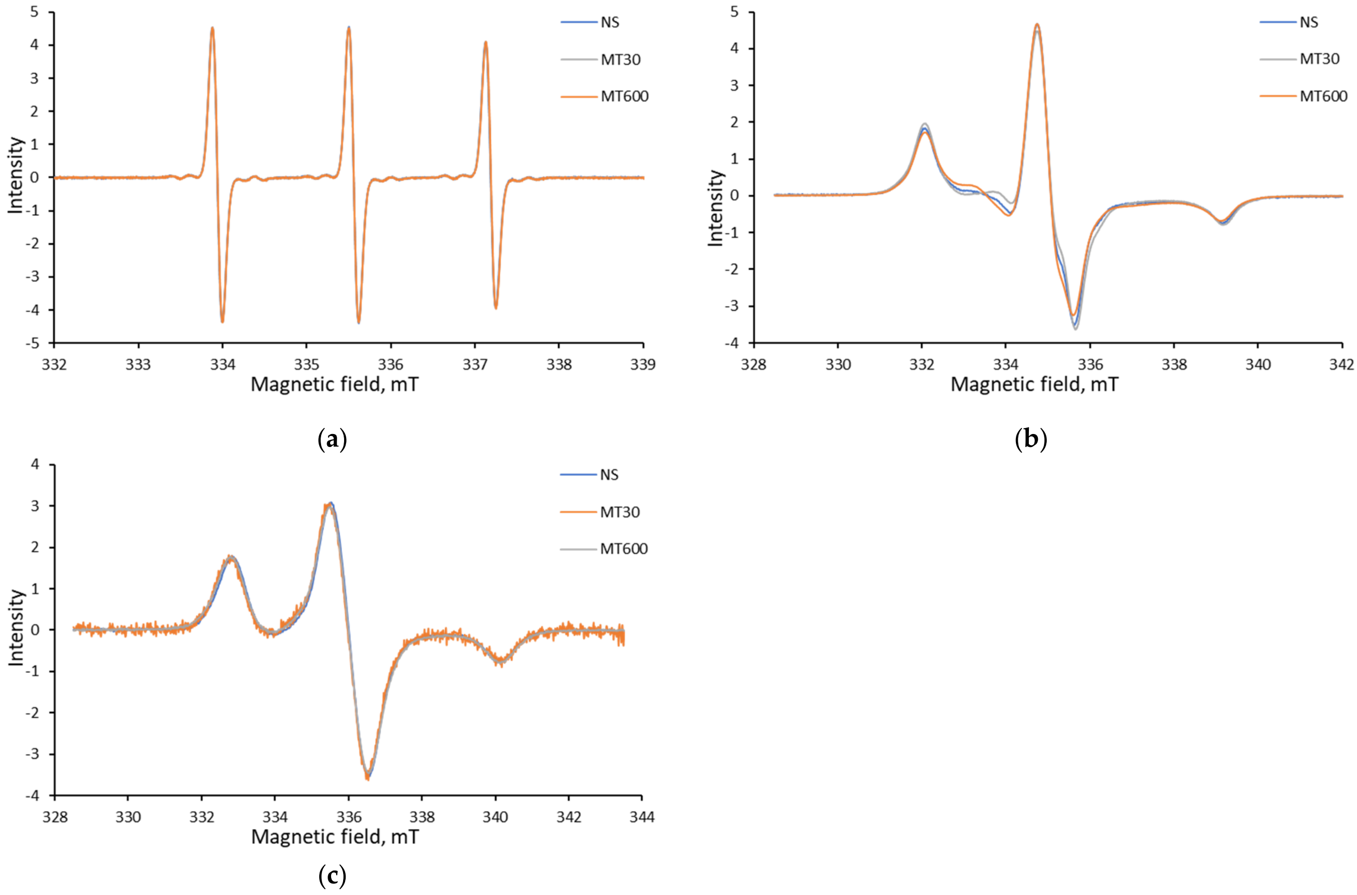

Figure S3. The characteristic EPR spectra of 3-carboxy-PROXYL nitroxide radical for corn starch: in the liquid state (a); in the film at room temperature (b); and the stabilized spectrum at –196°C (c).

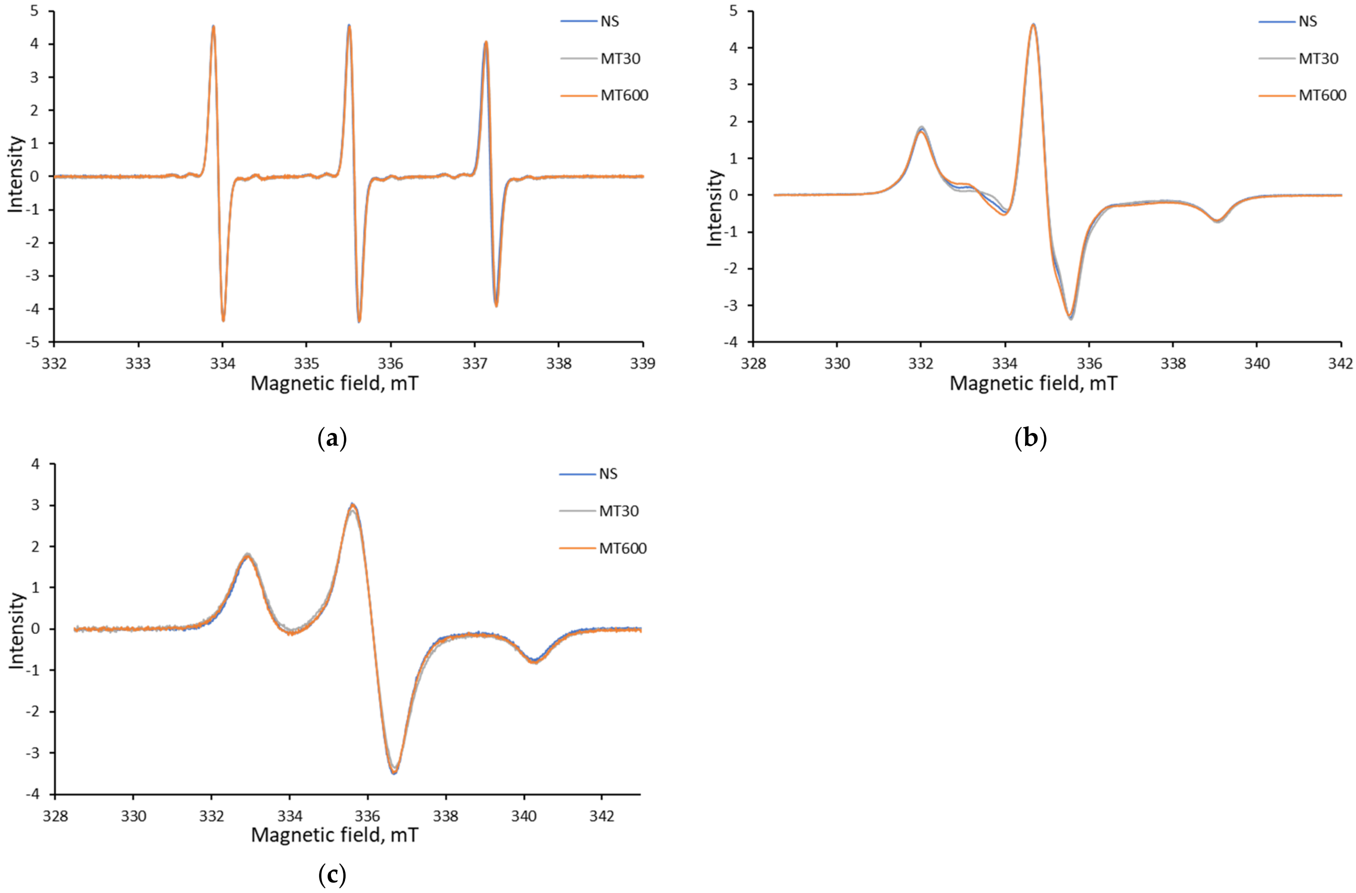

Figure S4. The characteristic EPR spectra of 3-carboxy-PROXYL nitroxide radical for tapioca starch: in the liquid state (a); in the film at room temperature (b); and the stabilized spectrum at –196°C (c).

Table S1. The key physicochemical characteristics of films based on native starch and starch with different disordering degrees (without 3-carboxy-PROXYL added).

Starting material	Type	Crystallinity index, %	Film thickness, μm
	Corn starch-based film		
NS[1]	A-type	19 ± 1	118 ± 8
MT30[2]	A-type	11 ± 1	85 ± 3
MT600[3]	B-type	22 ± 1 [a]	89 ± 12 [b]
	Potato starch-based film		
NS	B-type	25 ± 1	143 ± 8
MT30	B-type	17 ± 1	88 ± 4
MT600	B-type	26 ± 2 [a]	91 ± 6 [b]
	Tapioca starch-based film		
NS	C-type	11 ± 1	136 ± 14
MT30	C-type	8 ± 1 [a]	75 ± 15
MT600	B-type	19 ± 1	70 ± 5 [b]

Data are presented as mean ± SD. [1] NS – native starch; [2] MT30 – starch after mechanical treatment for 30 s; [3] MT600 – starch after mechanical treatment for 600 s. [a] No significant differences compared to NS samples (p-value ≥ 0.05). [b] No significant differences compared to MT30 samples (p-value ≥ 0.05).
